# Possible Existence of Cochlear Synaptopathy in Patients Completely Recovered from Idiopathic Sudden Sensorineural Hearing Loss

**DOI:** 10.3390/jcm11030875

**Published:** 2022-02-07

**Authors:** Hee Won Seo, Seung Yeol Lee, Hayoung Byun, Seung Hwan Lee, Jae Ho Chung

**Affiliations:** Department of Otolaryngology—Head and Neck Surgery, College of Medicine, Hanyang University, Seoul 04763, Korea; vngufb@naver.com (H.W.S.); seungyeollee88@gmail.com (S.Y.L.); hayoung.byun.md@gmail.com (H.B.); shleemd@hanyang.ac.kr (S.H.L.)

**Keywords:** sudden hearing loss, synaptopathy, hidden hearing loss, auditory brainstem response

## Abstract

Cochlear synaptopathy refers to a subclinical hearing pathology which could potentially explain hearing difficulties within the normal hearing threshold; it is also called “hidden hearing loss”. We hypothesized that a temporary threshold shift in sudden sensorineural hearing loss (ISSNHL) also affects the function in the synapse. The aim of the study was to evaluate the presence of cochlear synaptopathy in patients who had completely recovered from unilateral SSNHL Nineteen patients who had completely recovered from ISSNHL from January 2018 to June 2021 were assessed. Complete recovery was established by pure tone audiometry (PTA) 3 months after treatment, according to the American Academy of Otolaryngology–Head and Neck Surgery criteria. Subjects completed the pure tone audiometry, speech audiometry and auditory brain stem response (ABR) test, and completed a questionnaire regarding hearing loss after hearing recovery. The ABR amplitudes of wave I and wave V, and the ratio of wave I/V of both ears (recovered side and healthy side) were assessed. A visual analog scale (VAS) and a hidden hearing loss questionnaire were used to evaluate subjective hearing difficulty. The ABR waves I of the recovered ears had a significantly lower amplitude (*p* = 0.002) than those of the healthy side, whereas there was no difference in wave V (*p* = 0.985) or in the ratio of wave I/V (*p* = 0.107). Some patients still felt mild hearing difficulty although their PTA results were normal, but there was no clear relationship between the VAS score, wave I amplitude and speech recognition scores. The present findings point to the possible existence of cochlear synaptopathy in ears that have completely recovered from unilateral sudden sensorineural hearing loss. We suggest that the causes of cochlear synaptopathy and of idiopathic sudden hearing loss may have something in common.

## 1. Introduction

Cochlear synaptopathy refers to a subclinical hearing pathology which could potentially explain hearing difficulties within the normal hearing threshold, and is also called “hidden hearing loss” [1]. Patients with cochlear synaptopathy often complain of tinnitus or difficulty in recognizing speech in a noisy environment even though they have normal hearing [2]. The term hidden hearing loss was introduced by Schaette and McAlpine when they found that the amplitude of auditory brainstem response (ABR) wave I was reduced in tinnitus patients with normal audiograms [3]. Its etiology is unknown, but noise exposure, aging and ototoxic drugs are considered risk factors [2,4].

Animal studies have revealed a decrease in the number of ribbon synapses between inner hair cells and auditory nerve fibers as a result of repetitive exposure to noise, or aging, and such cochlear synaptopathy is considered a main cause of hidden hearing loss [5,6]. It occurs without damage to the hair cells causing permanent hearing loss, and leads to a temporary threshold shift (TTS), which is a reversible change in hearing threshold [7]. Although hearing recovery from TTS has been assumed to indicate the reversal of inner ear damage, the loss of ribbon synapses could be related to the recovery course of temporary threshold shift, even to the point of full hearing recovery [8].

Idiopathic sudden sensorineural hearing loss (ISSNHL) is defined as a sudden increase of 30dB or more in the hearing threshold across three or more consecutive frequencies, and it considered an emergent condition needing prompt management [9]. It is known that 21–28% of patients with sudden sensorineural hearing loss (SSNHL) recover completely after medical treatment [10,11,12]. Since the etiology of SSNHL is unknown [13], it is not yet clear what changes occur in the auditory nerve fibers or cochlea after hearing recovery. In addition, patients whose hearing threshold has completely recovered complain of perceptual difficulties, such as in speech recognition. In this regard, we hypothesized that recovering from ISSNHL is similar to experiencing prolonged TTS which is related to cochlear synaptopathy.

The aim of the present study was to investigate the possibility of cochlear synaptopathy by measuring ABR amplitude in patients who had recovered completely from unilateral SSNHL.

## 2. Materials and Methods

### 2.1. Patients and Study Design

Among consecutive ISSNHL patients from January 2018 to June 2021, ISSNHL patients with complete recovery were selected and assessed for hidden hearing loss. Based on the 2019 American Academy of Otolaryngology–Head and Neck Surgery (AAO–HNS) guidelines, SSNHL was defined as hearing loss of ≥30 dB at 3 consecutive frequencies within 3 days without an identifiable cause. All patients were treated with uniform management protocol of high-dose oral steroid and salvage intratympanic steroid injection. Complete recovery was defined as hearing returning to within 10 dB of the contralateral (healthy) ear at 3 months after treatment [14]. In addition, patients with previous contralateral hearing loss of ≥30 dB, or those diagnosed with Meniere’s disease, showing fluctuating hearing loss or retrocochlear lesions including vestibular schwannoma were not included in the analysis.

Finally, a total of 19 patients were included. After complete hearing recovery, identified by pure tone audiometry (PTA), patients underwent word recognition test and ABR test. Additionally, subjective hearing status after hearing recovery was evaluated via a hearing questionnaire. In addition, medical records including basic characteristics of the patients, underlying diseases, associated symptoms and treatment methods, were also reviewed.

### 2.2. Ethical Issues

Written informed consent was obtained from all patients, and the investigation was approved by the Institutional Review Board (IRB) of Hanyang University Guri Hospital and performed in accordance with the Declaration of Helsinki and Good Clinical Practice guidelines (IRB FILE No: 2021-10-002).

### 2.3. Audiometric Evaluation

**Figure 1:**
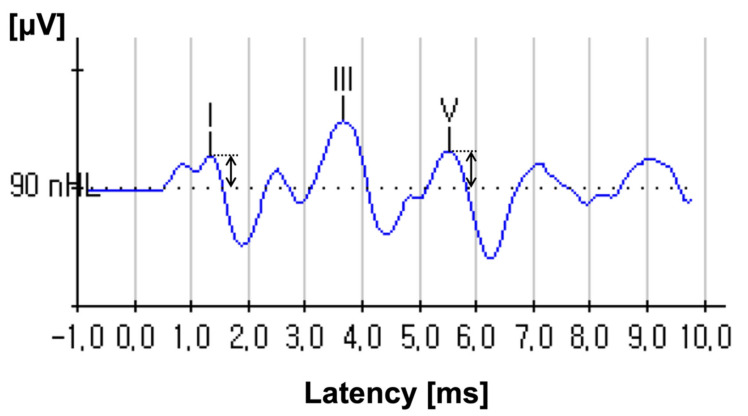
An auditory brainstem response (ABR) wave in response to a 90 dB nHL click. The vertical arrows show how the amplitudes of waves I and V are measured.

All eligible patients underwent PTA, a word recognition test and ABR tests after hearing recovery. PTA was performed in the frequency range of 250–8000 Hz, and thresholds were recorded and calculated as the average thresholds at frequencies of 0.5, 1, 2 and 4 kHz. The word recognition score (WRS) was evaluated using the words in Korean Standard Monosyllabic Word Lists for adults (KS-MWL-A). A total of 50 words were randomly presented to listeners at most comfortable level of loudness and the percentage of correct responses were calculated. According to WRS, we divided the subjects into WRS of 100% group and WRS of less than 100% group. The ABR test was performed in a soundproofed room using Biologic Navigator Pro (Biologic System Corp., Mundelein, IL, USA). The test side ear was stimulated by clicks of 90 dB nHL intensity and 100 μs duration repeated at 13.30/s, with alternating polarity. Potentials were amplified with a gain of 100,000 using a low-pass filter of 300 Hz and high-pass filter of 3 kHz. The peaks of each wave were annotated manually by a single senior audiologist by averaging data from two trials. The noise level was kept at zero during the measurement process, and the sweep number was standardized at 1024. In addition, contralateral masking used at a 50 dB HL for counterbalance. The ABR measurements were concentrated on the peaks of wave I and wave V, and amplitude was defined as the vertical distance from the positive peak of each wave to the baseline (Figure 1) [15]. With the ABR results with post-recovery status, the wave I, V amplitude and wave I/V ratio were compared between the unaffected ear and the recovered ear.

### 2.4. Hearing Questionnaires

The hearing questionnaire was divided into two domains: the visual analogue scale (VAS) score and the hearing index. The VAS score used a 6-point scale, allowing patients to judge the level of hearing difficulty that they felt on a scale from 0 to 5.

(0) No hearing difficulty;
(1) Mild hearing difficulty;
(2) Moderate hearing difficulty;
(3) Moderate to severe hearing difficulty;
(4) Severe hearing difficulty;
(5) Cannot hear at all.

In addition, since there are no established guidelines for diagnosing hidden hearing loss, the authors developed four questions based on the signs that may appear in hidden hearing loss, and used them as a hearing index. The hearing index was calculated by adding up the scores (0 to 4) for which the patient answered ‘yes’ to the four following questions.

(1) Do you have trouble hearing when there is noise in the background?
(2) Do you have a problem hearing on the telephone?
(3) Is it difficult for you to follow a conversation when people talk at once?
(4) Do you feel people you are talking to seem to mumble?

### 2.5. Statistical Analysis

Statistical analyses were performed with Statistical Package for the Social Sciences (SPSS) for Windows 26.0 (IBM Corp., Armonk, NY, USA). Continuous variables were described by means ± standard deviations and data normality was confirmed with a p–p plot. Paired Student’s *t*-test were used to compare them, and categorical variables were expressed as frequencies and percentages. A two-tailed *p*-value < 0.05 was considered statistically significant.

## 3. Results

### 3.1. Patient Characteristics

**Figure 2:**
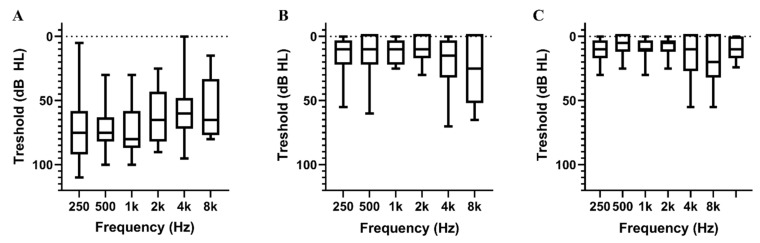
The mean pure tone threshold for each frequency: (**A**) pure tone threshold of the affected ear at initial presentation, (**B**) pure tone threshold after recovery, (**C**) unaffected side. The line with dots meant the 0 dB HL.

Of the 19 patients who completely recovered from SSNHL, 10 were male (52.6%) and the mean age was 45.7 ± 16.3 years. A total of 2 (10.5%) patients had hypertension, 5 (26.3%) had diabetes mellitus and 5 (26.3%) had cardiovascular disease. SSNHL was present on the right and left side in 31.6% (6 patients) and 68.4% (13 patients) of the patients, respectively. Associated symptoms included vertigo (15.8%), tinnitus (89.5%) and ear fullness (78.9%). The mean time from onset of symptoms to treatment was 3.5 ± 2.4 days. A total of 17 (89.5%) patients were treated with a high dose of steroids, of which 13 (68.4%) also received an intratympanic steroid injection. A total of 2 (10.5%) patients only received the intratympanic steroid injection because systemic steroids were contraindicated. The mean initial hearing threshold of the affected ear was 65.7 ± 17.8 dB, which was restored to 12.6 ± 7.8 dB after treatment. The hearing thresholds of the affected ears at each frequency before and after treatment are shown in Figure 2A,B. The mean hearing threshold of the healthy ear was 9.3 ± 7.2 dB (Table 1 and Figure 2C).

### 3.2. ABR Amplitudes

All patients underwent ABR testing after hearing recovery, and the amplitudes of wave I and wave V were obtained. The mean amplitude of wave I was 0.16 ± 0.10 μV on the recovered side, significantly lower than on the healthy side (0.22 ± 0.13 μV; *p* = 0.002). On the other hand, the mean ABR amplitude of wave V was 0.21 ± 0.08 μV on the recovered side and 0.24 ± 0.10 μV on the healthy side (*p* = 0.985). In addition, the wave amplitude ratio I/V was 0.94 ± 0.73 on the recovered side and 0.97 ± 0.66 on the healthy side (*p* = 0.107) (Table 2).

### 3.3. Hearing Questionnaire

**Figure 3:**
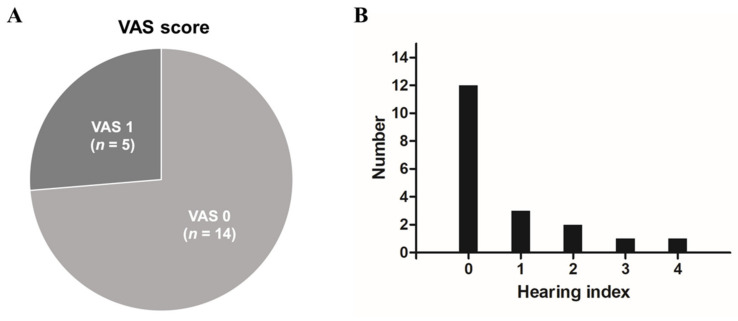
Visual analogue scale for hearing difficulty and hearing index (**A**) Pie chart showing the patient distribution according to the visual analogue scale (VAS) score. (**B**) The number of patients according to the hearing index score.

Figure 3 shows the results of the hearing questionnaire. In terms of subjective symptoms seen from the VAS score, 14 patients (73.7%) answered with a score of 0, which is normal hearing. On the other hand, 5 patients (26.3%) scored 1, indicating that they still felt mild hearing loss even though the pure tone threshold was normal. However, none of the patients complained of severe hearing loss, with a VAS score of 2 or more. When asked through the hearing index about situations in which they felt discomfort in daily life, 12 patients (63.2%) did not complain of discomfort in any of the situations, whereas 3 patients (15.8%) responded that they felt discomfort in one situation, 2 patients (10.5%) in 2 situations, 1 patient (5.3%) in 3 situations and 1 patient (5.3%) in all situations.

### 3.4. Association between Wave I Amplitude and Other Parameters

**Figure 4:**
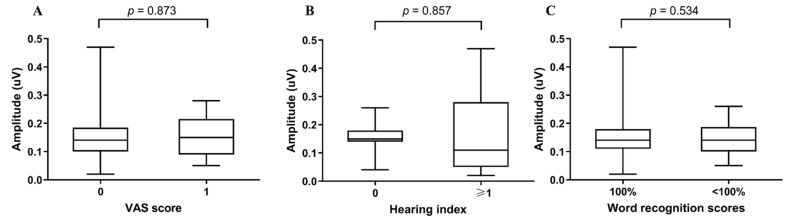
Box and whisker plots of ABR amplitude of wave I according to (**A**) VAS score, (**B**) hearing index and (**C**) word recognition scores.

When we analyzed the amplitudes of ABR wave I according to the VAS score, the mean amplitude of ABR wave I in the VAS 0 group was 0.16 ± 0.11 μV, which was not significantly different from the mean amplitude of the VAS 1 group, 0.15 ± 0.11 μV (*p* = 0.873). According to the hearing index, the mean amplitude of ABR wave I in the 0-point group without any discomfort was 0.15 ± 0.05 μV, which again was not statistically different from the amplitude in the group complaining of at least one discomfort (0.17 ± 0.16 μV; *p* = 0.857). In addition, the amplitude of the ABR I wave in the word recognition score of the 100% group and less than the 100% group did not show any statistical significance (Figure 4).

## 4. Discussion

In previous studies, cochlear synaptopathy has been considered a major mechanism of hidden hearing loss. Our intention in this study was to evaluate the possibility of cochlear synaptopathy in patients who have completely recovered from SSNHL. The results of the study can be summarized as follows. (1) The amplitude of ABR wave I on the recovered side was significantly lower than that on the healthy side (*p* = 0.002), whereas the amplitude of ABR wave V and the ratio of amplitudes I/V were not significantly different. (2) According to the VAS score and hearing index, 26.3% (5/19) and 36.8% (7/19) of the patients, respectively, complained of mild hearing discomfort even after complete recovery, but this was not associated with a decrease in the amplitude of ABR wave I.

The cause of hearing impairment can involve a defect of the cochlea, auditory nerve, or various neurotransmitters in synapses. Defects of the outer hair cells, which are considered the main cause of sensorineural hearing loss, disrupt cochlear amplification [16]. On the other hand, since the inner hair cells excite the sensory fibers of the cochlear nerve by releasing neurotransmitters to the synapses, damage to this area interferes with the conduction of auditory information or sound encoding. Exposure to noise causes excessive release of the neurotransmitter glutamate from presynaptic terminals, which leads to overexcitation and massive sodium influx into spiral ganglion neurons, leading to osmotic swelling and eventually damage to the nerve terminals [17]. In mice, it has been shown that the number of synaptic ribbons began to decrease without hair cell loss after 24 h of noise exposure and did not recover until 8 weeks [5]. Additionally, in a study observing cochlear synaptopathy after single blast exposure to chinchillas to peak pressures from 160–175 dB SPL, TTS of 40 dB or more, and synaptic loss of 20–45%, were observed [18]. In another study in mice, synapse loss increased by 50% as the noise dose increased, and this cochlear synaptopathy led to a decrease in the amplitude of ABR wave I [19].

The ABR is a recording of the electric potential generated by activation of the auditory nerve and brainstem through the scalp electrode. Wave I represents the compound action potential of the distal cochlear nerve in contact with the inner hair cell, and wave V represents the potentials generated by the auditory brainstem [20]. Therefore, a decrease in the amplitude of ABR wave I implies impairment of the inner hair cell, auditory nerve fiber, or the synapse between the two. In cochlear synaptopathy, the amplitude of ABR wave I decreases, whereas the amplitude of ABR wave V, the brainstem response, is maintained, or increases as a compensatory response, so the ratio of amplitudes, I/V, may also decrease [7,21]. Since in human studies, unlike animal experiments, synaptic loss cannot be directly observed, a decrease in amplitude of ABR wave I or the I/V ratio can be used as important evidence of cochlear synaptopathy.

The presumed causes of SSNHL include autoimmune inner ear diseases, viral infections, vascular disorders, ototoxic medication, genetic disorders, trauma, and acoustic tumors, but in most cases, the etiology is uncertain [14,22]. As opposed to noise-induced hearing loss, where one can study cochlear synaptopathy in noise-exposed animal models, it is impossible to design animal models to study the pathophysiology of SSNHL. Therefore, the present study aimed to investigate the possibility of cochlear synaptopathy by measuring the amplitude of ABR in patients who had recovered from SSNHL. Since most patients visited the outpatient clinic for the first time after the onset of SSNHL so that their hearing threshold before the onset of SSNHL could not be known, the healthy contralateral ear was used as control, and a decrease in the amplitude of ABR wave I was observed in the recovered ear compared with the healthy ear. This suggests that cochlear synaptopathy may remain after recovery from SSNHL, and that the causes of SSNHL and of cochlear synaptopathy may have something in common. However, there was no difference in the amplitude of ABR wave V on the recovered side compared with the healthy side, though both were rather low, and the I/V ratio was also not significantly different between the two sides. We suppose that wave V, the brainstem response, is less sensitive to cochlear synaptopathy than wave I.

In this study, we found no correlation between amplitude of ABR wave I and subjective symptoms obtained via hearing questionnaire. This means that some patients may experience perceptual difficulty but have a normal amplitude of ABR wave I, or that other patients may feel they have normal hearing but show a decrease in the amplitude of ABR wave I, implying the possibility of cochlear synaptopathy. This may be because patients perceive sounds from both sides, and the contralateral hearing is normal. In addition, it is thought that subjective symptoms are expressed differently depending on the individual, because there is a complex reciprocal relationship between the peripheral and central auditory nervous systems in the auditory cognitive process.

The present study has several limitations. First, only a small number of patients were included in this study because we included only patients who had completely recovered from SSNHL. In addition, since there was no established tool for diagnosing hidden hearing loss, the authors investigated subjective symptoms through a self-designed questionnaire. Studies with a larger number of patients and a more detailed questionnaire are needed. Another limitation is the possibility that comparing the ABR amplitudes on the affected and contralateral sides may not be accurate because the amplitudes of the ABR wave on the affected side before the onset of hearing loss are unknown. Finally, further studies are required using electrocochleography or hearing in noise test, the alternative diagnostic tools for cochlear synaptopathy. Nevertheless, the present study has strength in that it is the first to demonstrate the possibility of cochlear synaptopathy in SSNHL.

## 5. Conclusions

The decrease in amplitude of the ABR wave I in ears that have completely recovered from SSNHL suggests that cochlear synaptopathy may be involved in the pathogenesis of SSNHL.

## Figures and Tables

**Table 1:** Demographic and clinical characteristics of the study population.

Variable	Patients *n* = 19
Sex (number, %)	
Male/Female	10 (52.6%)/9 (47.4%)
Age (years, mean, (range))	45.7 ± 16.3, (16–64)
Underlying disease (number, %)	
Hypertension	2 (10.5%)
Diabetes mellitus	5 (26.3%)
Cardiovascular disease	5 (26.3%)
Affected side	
Right/Left	6 (31.6%)/13 (68.4%)
Associated symptom (number, %)	
Vertigo	3 (15.8%)
Tinnitus	17 (89.5%)
Ear fullness	15 (78.9%)
Onset of treatment (days)	3.5 ± 2.4
Initial hearing	
Pure tone threshold (dB)	65.7 ± 17.8
Word recognition score (%)	34.0 ± 31.3
Hearing level after treatment	
Pure tone threshold (dB)	12.6 ± 7.8
Word recognition score (%)	97.3 ± 3.9
Hearing level of unaffected side	
Pure tone threshold (dB)	9.3 ± 7.2
Word recognition score (%)	99.6 ± 1.2
Treatment method (number, %)	
High dose steroid	4 (21.1%)
Oral steroid + Intratympanic steroid	13 (68.4%)
Intratympanic steroid only	2 (10.5%)

**Table 2:** Comparison of the auditory brainstem response parameters between the recovered side and the healthy side.

	Wave I (μV)	Wave V (μV)	Wave I/V Ratio
Recovered side	0.16 ± 0.10	0.21 ± 0.08	0.94 ± 0.73
Healthy side	0.22 ± 0.13	0.24 ± 0.10	0.97 ± 0.66
*p* value *	0.002	0.985	0.107

* Paired *t*-test.

## Data Availability

Anonymized data will be shared with any qualified investigators on request.

## References

[1] Marmel F., Cortese D., Kluk K. (2020). The ongoing search for cochlear synaptopathy in humans: Masked thresholds for brief tones in Threshold Equalizing Noise. Hear. Res..

[2] Plack C.J., Barker D., Prendergast G. (2014). Perceptual consequences of “hidden” hearing loss. Trends Hear..

[3] Schaette R., McAlpine D. (2011). Tinnitus with a normal audiogram: Physiological evidence for hidden hearing loss and computational model. J. Neurosci..

[4] Kohrman D.C., Wan G., Cassinotti L., Corfas G. (2020). Hidden Hearing Loss: A Disorder with Multiple Etiologies and Mechanisms. Cold Spring Harb. Perspect. Med..

[5] Kujawa S.G., Liberman M.C. (2009). Adding insult to injury: Cochlear nerve degeneration after “temporary” noise-induced hearing loss. J. Neurosci..

[6] Kujawa S.G., Liberman M.C. (2015). Synaptopathy in the noise-exposed and aging cochlea: Primary neural degeneration in acquired sensorineural hearing loss. Hear. Res..

[7] Shi L., Chang Y., Li X., Aiken S., Liu L., Wang J. (2016). Cochlear Synaptopathy and Noise-Induced Hidden Hearing Loss. Neural Plast..

[8] Seo J.K., Lim H.W., Park H.J., Pak J.H., Chung J.W. (2013). Changes of Cochlear Nerve Terminals after Temporary Noise-Induced Hearing Loss. Korean J. Otorhinolaryngol. Head Neck Surg..

[9] Rauch S.D. (2008). Clinical practice. Idiopathic sudden sensorineural hearing loss. N. Engl. J. Med..

[10] Edizer D.T., Çelebi Ö., Hamit B., Baki A., Yiğit Ö. (2015). Recovery of Idiopathic Sudden Sensorineural Hearing Loss. J. Int. Adv. Otol..

[11] Rhee T.M., Hwang D., Lee J.S., Park J., Lee J.M. (2018). Addition of Hyperbaric Oxygen Therapy vs Medical Therapy Alone for Idiopathic Sudden Sensorineural Hearing Loss: A Systematic Review and Meta-analysis. JAMA Otolaryngol. Head Neck Surg..

[12] Mirian C., Ovesen T. (2020). Intratympanic vs. Systemic Corticosteroids in First-line Treatment of Idiopathic Sudden Sensorineural Hearing Loss: A Systematic Review and Meta-analysis. JAMA Otolaryngol. Head Neck Surg..

[13] Seo H.W., Chung J.H., Byun H., Lee S.H. (2021). Vestibular mapping assessment in idiopathic sudden sensorineural hearing loss. Ear Hear..

[14] Chandrasekhar S.S., Tsai Do B.S., Schwartz S.R., Bontempo L.J., Faucett E.A., Finestone S.A., Hollingsworth D.B., Kelley D.M., Kmucha S.T., Moonis G. (2019). Clinical Practice Guideline: Sudden Hearing Loss (Update). Otolaryngol. Head Neck Surg..

[15] Kamerer A.M., Neely S.T., Rasetshwane D.M. (2020). A model of auditory brainstem response wave I morphology. J. Acoust. Soc. Am..

[16] Dallos P. (2008). Cochlear amplification, outer hair cells and prestin. Curr. Opin. Neurobiol..

[17] Moser T., Predoehl F., Starr A. (2013). Review of hair cell synapse defects in sensorineural hearing impairment. Otol Neurotol.

[18] Hickman T.T., Smalt C., Bobrow J., Quatieri T., Liberman M.C. (2018). Blast-induced cochlear synaptopathy in chinchillas. Sci. Rep..

[19] Fernandez K.A., Guo D., Micucci S., De Gruttola V., Liberman M.C., Kujawa S.G. (2020). Noise-induced Cochlear Synaptopathy with and Without Sensory Cell Loss. Neuroscience.

[20] Eggermont J.J. (2019). Auditory brainstem response. Handb. Clin. Neurol..

[21] Kikidis D., Vardonikolaki A., Zachou Z., Razou A., Pantos P., Bibas A. (2020). ABR findings in musicians with normal audiogram and otoacoustic emissions: Evidence of cochlear synaptopathy?. Hear. Balance Commun..

[22] Byun H., Chung J.H., Lee S.H. (2020). Clinical implications of posterior semicircular canal function in idiopathic sudden sensorineural hearing loss. Sci. Rep..

